# Development of the Warwick Axial Spondyloarthritis faTigue and Energy questionnaire (WASTEd)—a new patient-reported outcome measure

**DOI:** 10.1093/rap/rkac027

**Published:** 2022-04-04

**Authors:** Nathan A Pearson, Elizabeth Tutton, Jane Martindale, George Strickland, Jean Thompson, Jonathan C Packham, Paul Creamer, Kirstie L Haywood

**Affiliations:** 1 Warwick Research in Nursing, Warwick Medical School, University of Warwick, Coventry; 2 Kadoorie, Oxford Trauma and Emergency Care, Nuffield Department of Orthopaedics, Rheumatology and Musculoskeletal Sciences, University of Oxford; 3 John Radcliffe, Oxford University Hospitals NHS Foundation Trust, Oxford; 4 Rheumatology, Wrightington, Wigan and Leigh NHS Foundation Trust, Wigan; 5 Faculty of Health and Social Care, Edge Hill University, Ormskirk; 6 Academic Unit of Population and Lifespan Sciences, University of Nottingham, Nottingham; 7 Haywood Academic Rheumatology Centre, The Haywood Hospital, Stoke-on-Trent; 8 Rheumatology Department, North Bristol NHS Partnership Trust, Bristol, UK

**Keywords:** Fatigue, energy, axial spondyloarthritis, outcome assessment, measurement, qualitative, patient-reported outcomes, co-production, active collaboration

## Abstract

**Objective:**

The aim was to co-produce and test a potential new patient-reported outcome measure (PROM), the Warwick Axial Spondyloarthritis faTigue and Energy questionnaire (WASTEd), providing vital qualitative confirmation of conceptual relevance, clarity and acceptability.

**Methods:**

Informed by measurement theory, we collaborated with patient partners throughout a three-stage, iterative process of PROM development. In stage 1, informed by patient interviews, reviews exploring patients’ fatigue experiences and existing PROMs of fatigue, an initial measurement framework of axial spondyloarthritis (axSpA) fatigue and energy and candidate items were defined. In stage 2, the relevance and acceptability of the measurement framework and candidate items were assessed qualitatively by focus group participants. In stage 3, patients participated in pre-testing interviews to assess item comprehensiveness, relevance, acceptability and comprehensibility.

**Results:**

Stage 1 informed the development of an initial five-domain measurement framework with 59 candidate items. In stage 2, five patients and seven health-care professionals participated in four focus groups to derive a 40-item model of fatigue and energy. Collaborative engagement with patient research partners supported refinement of questionnaire structure and content further. Pre-testing with ten patients across two interview rounds in stage 3 produced a four-domain, 30-item long-form questionnaire.

**Conclusion:**

An active collaboration with patients and health-care professionals has supported the co-production of a potential new PROM of axSpA fatigue, underpinned by strong evidence of face and content validity. The WASTEd extends the assessment of fatigue beyond severity, highlighting the importance of symptom frequency, energy and self-management. Future research will involve psychometric evaluation, supporting item reduction, structural refinement and confirmation of PROM validity.

Key messages

- Single-item assessments of fatigue severity do not fully capture patient experience of fatigue.
- Qualitative research informed the development of a multidomain measurement framework of energy and fatigue.
- The co-produced draft measure of fatigue and energy (WASTEd) has strong face and content validity.

## Introduction

Axial spondyloarthritis (axSpA) is a progressive, disabling rheumatic disease, often beginning in early adulthood [1, 2]. AxSpA typically advances slowly, often leading to an insidious decline in quality of life and in physical and social functioning [3]. Although pain, stiffness and reduced mobility are cardinal features [1, 2], fatigue is a major concern to patients, with ≤75% experiencing severe fatigue [4].

Patients describe wide-ranging adaptations attempting to mitigate the impact of fatigue on their daily life and social activities [5, 6]. Growing recognition of the importance of fatigue resulted in its inclusion in updated axSpA outcome reporting guidance [7], with the recommended assessment being a single-item measure of fatigue severity (taken from the BASDAI) [8]. However, significant limitations associated with single-item assessment include the inability to detail the nuances of fatigue experience/impact and a risk of overlooking some patients experiencing major fatigue-related impairment [4]. A recent systematic review of the quality and acceptability of single and multi-item, fatigue-specific patient-reported outcome measures (PROMs) in axSpA patients highlights further methodological inadequacies, including poor conceptual underpinning and limited relevance to patients’ experience of fatigue [9]. No measures involved patients as collaborative research partners in PROM development. The review concluded that existing measures were likely to underestimate the significant impact of fatigue in axSpA. It is therefore unsurprising that health professionals often overlook the fatigue experienced by axSpA patients [5].

A patient-derived, multi-item PROM specific to the experience of axSpA fatigue would be invaluable in evidencing the significant impact of fatigue on patients’ lives, highlighting their unmet needs to health-care professionals and supporting the provision of targeted and timely care. The active engagement with patients in rigorous qualitative research seeks to ensure that the outcomes that really matter to patients are included in PROM development, enhancing face and content validity, relevance and acceptability [10–14]. This study describes the initial qualitative stages in the development of a new measure of fatigue in axSpA.

## Methods

**Fig. 1:**
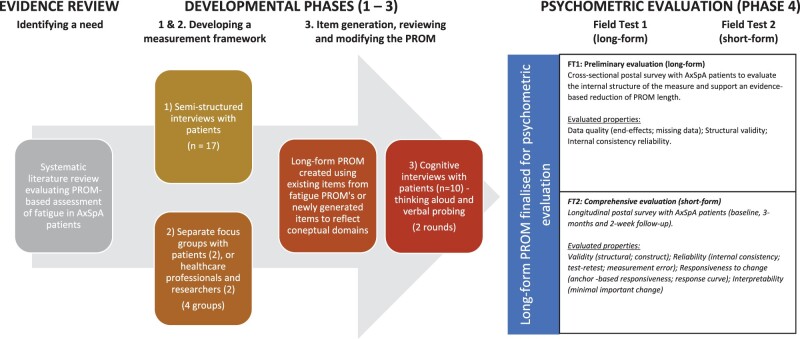
A flowchart to illustrate the development process of the Warwick Axial Spondyloarthritis faTigue and Energy questionnaire (WASTEd) PROM: Patient-Reported Outcome Measure.

This study is part of a five-stage project and describes a three-stage qualitative process to establish a potential new axSpA fatigue-specific PROM (Fig. 1). Study methods involved semi-structured interviews with patients (stage 1), focus groups with patients and health-care professionals (stage 2), and cognitive and pre-test interviews (stage 3). Working in collaboration with patients as research partners (PRPs) throughout all stages (Supplementary Table S1, available at *Rheumatology Advances in Practice* online), an iterative process of item development and refinement is described. National Health Service ethical approval was granted (REC reference: 16/WM/0147), and written informed consent was obtained from participants in all stages.

Informed by international guidance [12, 13, 15], three qualitative development stages are described:

1. Development of a measurement framework. This step clarifies the measurement focus by identifying essential domains, subdomains and patient-important outcomes from the patient perspective.
2. Confirming the measurement framework and refining candidate items. The purpose is to refine the measurement framework and ensure that it covers essential content, enhancing relevance to patients and health-care professionals.
3. Pre-testing items and confirming content validity. This focuses on clarifying the comprehensiveness, relevance, acceptability and comprehensibility of the developing PROM through qualitative evaluation with patients.

### Stage 1: development of a measurement framework

A measurement framework provides structure for what should be measured, describing the overarching concept of health and anticipated relationships between domains and patient-important outcomes [14]. Qualitative research is essential to development, clarifying the essence of axSpA from the perspective of a patient [10, 11].

Qualitative semi-structured interviews, drawing on phenomenology as their methodological underpinning, were conducted with axSpA patients to explore their lived experiences of axSpA and fatigue. The interview study was conceived with PRPs who supported generation of the topic guide and the initial analysis. These interviews were re-analysed to inform the developing measurement framework [16]. A thematic analysis supported the extraction and grouping of patient-important outcomes and themes into domains and subdomains of similar or shared meaning [17]. An impact triad of severity, importance and self-management informed this process [18].

Working collaboratively and iteratively, members of the core research team (N.A.P., E.T., J.M., K.L.H. and J.C.P.) and PRPs (including G.S. and J.T.) reviewed the developing framework and potential questions (items). As part of our initial review of the quality and acceptability of PROMs used to assess fatigue in axSpA [9], we also sought additional reviews of fatigue PROMs across a range of conditions [19–21]. The item content of these existing measures of fatigue or energy were reviewed, and potential items judged relevant to the developing framework were identified and/or modified. Where necessary, new items were crafted from the qualitative data to reflect language used by patients [16].

### Stage 2: confirming the measurement framework and refining candidate items

Patients with confirmed axSpA [22] (age ≥18 years) and health-care professionals with experience of working with axSpA patients were invited to participate in separate focus groups. To explore the content, relevance and acceptability of the measurement framework and candidate items, focus group activities were structured into three parts (Table 1).

**Table 1:** Topics and example discussion points for the focus groups

Topic	Example discussion points
1. Discussing the findings of interviews, and the measurement framework of fatigue in axial spondyloarthritis (≤30 min)	What are your personal experiences of fatigue or tiredness? Do you recognize any similarities with the experiences we presented to you? Are there any differences?
	Have we included the most important concepts and outcomes about fatigue and tiredness in the framework?
2. Ranking what matters most to you (≤20 min)	Individual activity: write down your own ‘importance’ list
	Group activity: sharing your list with the group
	Group activity: group discussion about lists and discrepancies that appear
	Group activity: reaching a group agreement on the top six most important outcomes
3. Exploring the relevance and acceptability of the measurement framework and new items (≤50 min)	Which questions do you think work well? Why?
	Which questions do you think don’t work so well? Why?
	Are there any important questions that you think are missing?
	Do you think the measurement framework is thorough? Is anything missing?

Patients were recruited from rheumatology outpatient departments at three UK hospitals and purposively sampled for age, sex and disease duration. Professionals were identified through the Ankylosing Spondylitis Special Interest Group Northwest (ASSIGNw) network, rheumatology departments of participating sites, and known contacts. Informed consent was secured from all participants. All groups were moderated by N.A.P., co-facilitated by J.C.P. or J.M. and digitally audio recorded.

Focus groups followed a semi-structured format (Table 1). First, findings of the qualitative interviews were shared and discussed. The developing measurement framework was then presented, with each participant receiving a reference paper copy. Participants discussed the framework with reference to their own experiences, highlighting where important outcomes were missing. Second, participants ranked outcomes individually in order of importance. After group discussion, participants were asked to reach agreement on the most important outcomes. Finally, participants explored the relevance, acceptability and comprehensibility of candidate items. The group worked iteratively through the proposed items for each subdomain. N.A.P. and E.T. analysed the data thematically and deductively [17] after each group, drawing on the measurement framework and highlighting areas of resonance or dissonance with the proposed framework or items.

### Stage 3: pre-testing the items and confirming content validity

After stage two, a questionnaire was formatted with candidate items grouped into similar concepts. Two rounds of pre-testing interviews were conducted with patients (recruited as for stage 2) to explore item comprehensiveness, relevance, acceptability and comprehensibility [11, 12, 15]. These assessed whether participants could: understand items and formulate a response (comprehension); retrieve necessary information to enable a response (retrieval); determine the accuracy of retrieval (judgement); and select an appropriate response option (response mapping) [11, 15]. Techniques of thinking aloud and verbal probing were used to explore any problems with the questionnaire, item format or wording, to identify any remaining omissions in content and to confirm content validity [11]. Verbal probes were developed in collaboration with PRPs. The reading level (reading age, difficulty and accessibility) was assessed after each round using the Flesch Kincaid reading level [23].

#### Round 1

Items were grouped into sequential blocks of six items. After self-completing (while talking aloud) each block, the researcher questioned participants using verbal probes. These sought to elucidate whether there was a discernible, conceptual distinction between fatigue and energy at the point of item completion.

#### Round 2

Questionnaire completion and testing following the three-step test interview (TSTI) approach [24], as follows. First, to reflect usual questionnaire completion, participants were observed completing the full list of items, uninterrupted by the researcher. Participants were encouraged to think aloud during this activity. Their response behaviour was observed. Second, the participant’s experience of questionnaire completion was then explored by the researcher (verbal probes), supplemented by researcher observations, to check that items were understood as intended. Finally, a semi-structured debrief explored general questions about the questionnaire and item presentation; for example, layout, font sizing, white spacing etc.

#### Analysis of pre-testing interview data

Informed by international guidance [13–15] and a modified category set from the question appraisal system (QAS-99), an item assessment checklist was developed to ensure transparency in the decision-making process for item retention, modification or rejection (Table 2). Decisions were made collaboratively between research team members and PRPs.

**Table 2:** Item assessment checklist: identifying areas of concern when assessing items during pre-testing interviews [14, 26]

Categories	Subcategories	Challenges
1. Clarity	a. Wording	Lengthy question, poor grammar, complicated syntax, awkward to read
	b. Technical terms	Complex, lacks definition or clarity
	c. Vague	Multiple interpretations making response difficult
	d. Reference periods	Missing, poorly specified or conflicting
2. Assumptions	a. Inappropriate assumptions	Question inappropriately assumes something of the respondent
	b. Assumes constant behaviour	Fails to recognize that situations vary
	c. Double-barrelled	Asks more than one question of the respondent
3. Knowledge/memory	a. Knowledge	Respondent might not know an answer
	b. Recall	Respondent might not be able to recall the information
	c. Computation problem	Difficult mental calculations affecting responses
4. Sensitivity/bias	a. Sensitive content (general)	Embarrassing or private question for respondents
	b. Sensitive wording (specific)	Question should be worded to minimize sensitive responses
	c. Socially acceptable	Implied response by the question
5. Response categories	a. Open-ended	Difficult or inappropriate
	b. Mismatch	Do response options match the question?
	c. Technical terms	Complex, poorly defined or unclear language
	d. Vague	More than one interpretation for a given response option
	e. Overlapping	Response options are not distinct from one another (conflated)
	f. Missing	Categories that should be affirmed are missing data
	g. Illogical order	Categories should be logically ordered
6. Other problems	a. Other	Problem other than those defined
7. Variability in responses	a. Inactive response options	One or more response options not being endorsed by respondents

## Results

### Stage 1: development of a measurement framework of fatigue in axSpA

**Fig. 2:**
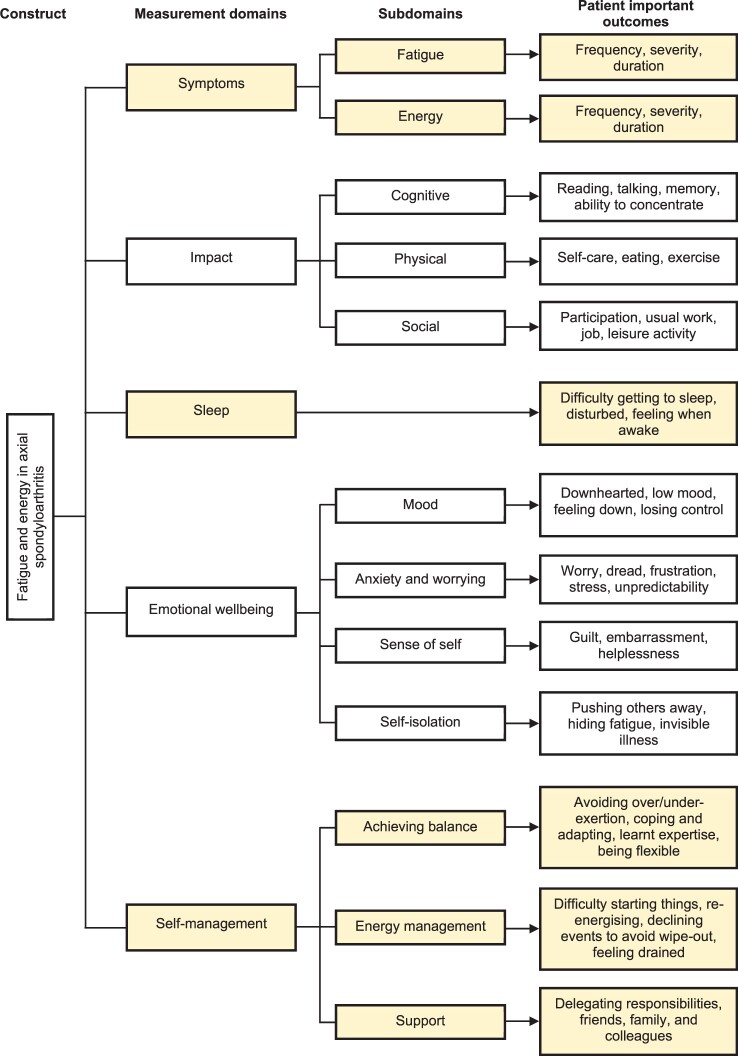
Stage 1: a working measurement framework of fatigue in axial spondyloarthritis

Development of the measurement framework was based on analysis of a prior study of 17 axSpA patients who participated in semi-structured, audio-recorded interviews (8 female: age range 22–72 years; disease duration range 1–41 years) [16]. Five domains were defined, reflecting 13 subdomains (Fig. 2): (1) symptoms: fatigue and energy; (2) impact: cognitive, physical and social; (3) sleep; (4) emotional wellbeing: mood, anxiety and worrying, sense of self and self-isolation; and (5) self-management: achieving balance, energy management and support.

Four reviews were identified describing the quality and acceptability of 26 fatigue-specific PROMs across a range of conditions, including RA, axSpA, cancer and Parkinson’s disease [9, 19–21]. A review of energy PROMs was not identified. However, items within the vitality subscale of the short-form 36-item health survey (SF-36) were reviewed [25]. From these PROMs, a total of 44 items were mapped to the developing measurement framework, capturing the conceptualized patient-important outcomes (Supplementary Table S2, available at *Rheumatology Advances in Practice* online). Fifteen items were newly crafted to reflect the remaining patient-important outcomes and complete coverage of the measurement framework.

The research team and PRPs reviewed all 59 items for comprehensiveness, relevance, item structure and language. Nineteen items were removed owing to repetition and unsuitable language (e.g. feeling ‘listless’ [26]). Phraseology was standardized for the 40 remaining items. Drawing on the findings from the patient interviews and guidance recommending shorter recall periods for variable, frequent symptoms, such as fatigue [14], a recall period of 1 week was selected.

Measurement theory guided consideration of two potential response scales [27]: numerical rating scale (NRS) or categorical, descriptive scales.

### Stage 2: confirming the measurement framework and refining items

A total of 41 patients and 30 health-care professionals were approached to participate in focus groups. Five patients [3 females; mean (range) age 55.6 (32–73) years; mean axSpA duration 24.2 years] participated across two groups (duration 2.5 and 3 h, respectively). Seven health-care professionals [4 females; mean (range) age 43.23 (30–54) years] who had experience of working with axSpA patients [median (range) 10 (1–33) years; 4 physiotherapists and 1 occupational therapist] participated across two groups (each 1.5 h).

All participants endorsed the proposed conceptualization of fatigue and energy in axSpA, confirming that nothing important was missing from the measurement framework or developing items. This provoked health-care professionals to comment on the limitations associated with the assessment of fatigue in clinical practice, ‘historically and even currently, the actual measures that we use don’t really factor in the fatigue elements … the fatigue isn’t asked about really’ (professional, group 2), welcoming the potential benefits of a questionnaire based on the developing measurement framework.

In each domain, discussions highlighted the distinctions between fatigue and energy, the importance of emotional wellbeing, challenges of sleep as a fatigue-related concept and benefit gained from a greater awareness of how patients cope with their fatigue.

#### Symptoms

Patients and professionals agreed on the importance of including items specific to fatigue and energy, with distinct questions pertaining to frequency and severity: ‘I would identify more with the lack of energy than fatigue’ (patient, group 1). However, fatigue duration was not recommended by either group.

#### Impact

The cognitive, physical and social impact of fatigue was discussed. Cognitive effects included difficulty in concentrating and recalling memories, although patients tended to link memory changes to ageing. Physical effects included difficulties with self-care, such as cooking and cleaning and exercising, owing to low energy. Social impact extended into work, caring responsibilities and personal life, such as leisure and social activities.

The importance of distinguishing between fatigue and energy was apparent in this domain, with patients agreeing that items on social activity were reflective of energy, rather than fatigue. Health-care professionals also highlighted the importance of financial impact. However, patients argued that financial implications were often associated with axSpA rather than specifically attributable to fatigue.

#### Sleep

This reflected challenges related to getting to sleep, staying asleep and waking feeling unrefreshed. Sleep as an important mediator of mood and fatigue (and therefore a person’s ability to function) was recognized by all participants. However, owing to the complexity and multidimensional nature of sleep, participants felt that it was an unhelpful item to include in a fatigue-specific measure; for example: ‘I just don’t sleep well; it’s nothing to do with my AS’ (patient, group 1). Participants agreed that sleep embraced too many variables to contribute meaningfully to an assessment of fatigue and energy and should be considered for removal after further assessment in stage 3 (pre-testing interviews).

#### Emotional wellbeing

A domain specific to the impact of fatigue on emotional wellbeing was endorsed strongly. Health-care professionals reflected on the failure of clinics to assess this aspect of emotional wellbeing appropriately with axSpA patients. However, terminology was challenged by one professional group as potentially beyond the scope of the WASTEd: ‘I like the word downhearted but not depressed. We need to take depressed out because … you shouldn’t diagnose yourself or someone else with depression [using this questionnaire]’ (professional, group 2).

#### Self-management

‘Self-management’ sought to address how patients achieved a balance in managing exertion/activity levels and making adaptions. Energy management was important, describing how decisions were made based on perceived energy expenditure, current stamina and methods to re-energise. ‘Support’ referred to how people delegate responsibilities and rely on others to continue with daily life and activities.

Health-care professionals welcomed the inclusion of separate domains: ‘with this we can tell whether those not coping [with fatigue] … and those who are managing …’ (professional, group 1). They identified a need for domain-level scoring to support care decision-making: ‘we want to be able to see change and use domain scores’ (professional, group 1).

All participants confirmed that a categorical, descriptive scale was easier to understand and respond to than a numerical rating scale. A 1 week recall period was supported. Some patients suggested that a longer recall period would capture previous fatigue experiences, whereas professionals were concerned that a longer recall period would have little clinical value.

After conclusion of the focus groups, a 32-item WASTEd was produced ready for consideration in pre-testing interviews. Four items captured symptoms, 11 impact, 2 sleep, 7 emotional wellbeing and 8 self-management. Practicable recommendations to improve the questionnaire, including scoring, scale type and presentation, are summarized in Table 3.

**Table 3:** Key recommendations for patient-reported outcome measure modification after focus groups

Point of interest	Recommendation
Presentation	Use size 14 font, recognizing that some patients might have issues with sight (e.g. iritis)
	Maximize white space between questions to enhance readability
Clinical use	Reduce the length of the final version for routine use in clinical practice, 20 items maximum
	Provide domain-level scoring, if psychometrically possible
Item content and language	Monitor sleep items in pre-testing interviews
	Modify energy items to be language neutral (e.g. ‘energy levels’ instead of ‘lack of energy’)
	Ensure items on social life use the concept of energy, not fatigue
	Provide definitions of fatigue and energy for clarity
	Change the term ‘depression’ to ‘downhearted’

### Stage 3: pre-testing the items and confirming content validity

Ten male patients [mean (range) age 52.8 (28–75) years; mean disease duration 18.5 years] participated in the pre-testing interviews: five in round 1 and five in round 2. Interviews were conducted in local rheumatology departments [mean (range) duration 80 (33–120) min].

The research team and PRP group reviewed the results after each round. Item modifications informed by round 1 were agreed before further testing in round 2. The checklist (Table 2) confirmed that most items were presented clearly and understood; no item was assigned more than two areas of concern. Minor modifications were suggested to improve question clarity or response option anchors (Supplementary Table S3, available at *Rheumatology Advances in Practice* online).

#### Round 1

Reading ease equated to 75.7, suggesting a reading age between 11 and 12 years [23]. From the 32 items, 13 remained unchanged. Concerns were raised across four of the seven assessment categories for 19 items. First, there were issues of clarity (17 items); for example, the item ‘How often have you felt drained?’ was amended to read, ‘How often have you felt drained of energy?’. In other examples, a suggestion to underline the word that was the focus of the question was proposed. For example, fatigue or energy. Second, there were concerns about assumptions (5 items). Items were perceived as being double-barrelled or interpreted differently by participants. For example, for the item, ‘Because of your energy levels, have you found it mentally difficult to start, or finish doing things?’, a participant responded, ‘if I could cross out start then I’d say not at all’ (R2). Item rephrasing was explored. Third, sensitivity (2 items): an item asking, ‘Has fatigue made you feel downhearted?’ elicited emotional responses from three participants, and in one interview, required a brief pause (R2, R3 and R5). Item rephrasing was explored. Fourth, regarding response options (3 items), anchors were refined.

The core research team and PRPs agreed on removal of three items and minor modifications; 29 items were retained for consideration in round 2.

#### Round 2

Reading ease equated to 69.7, suggesting a reading age of 13–15 years [23]. Minor issues were raised for 5 of the 29 items (Supplementary Table S4, available at *Rheumatology Advances in Practice* online). First, minor modifications were made to the language (3 items); for example, changing the item ‘preferred to be alone due to fatigue’ to read ‘need to be alone’, recognizing that this is not a person’s preference. Second, response anchor modification to improve meaning (2 items); for example, changing ‘always’ to ‘everyday’ as a response to the question, ‘How often have you felt fatigued?’. Third, one item about ‘coping with fatigue’, which was removed in round 1, was highlighted as important and ‘missing’. Modifications to the original item were explored between members of the core research team and PRPs. PRPs differentiated between the concept of managing fatigue (viewed as reflecting practical steps) and that of coping with fatigue (described as an internal process), providing important insight that supported item rewriting.

In response, minor changes to the identified items were made, and the ‘coping with fatigue’ item was re-introduced. This process produced a 30-item long-form version of the WASTEd ready for future statistical evaluation. The questionnaire is presented in Supplementary Data S1, available at *Rheumatology Advances in Practice* online.

## Discussion

The Warwick Axial Spondyloarthritis faTigue and Energy (WASTEd) questionnaire represents the first patient-derived, co-produced measure of fatigue and energy specific to the experience of patients with axSpA. Patients have made a substantial contribution to the development process, both as research partners and as participants, informing the co-production of a measurement framework and list of items that have resonance with their lived experience and an assessment of fatigue that is both understandable and useful. The engagement with clinicians in this process has ensured the clinical utility of the measure. Future application of the WASTEd in clinical and research settings will ensure that the patient’s experience of fatigue is communicated clearly in decision-making.

Historically, fatigue in axSpA has been assessed as a unidimensional construct of fatigue severity [8]. However, the results of the present study challenge the narrow focus of current assessment guidance and confirm fatigue as a complex, multidimensional experience, of which energy is an important and distinct component. Although increasingly recognized in other fields, including HIV [28, 29] and nephrology [30], this is the first time that the importance of energy has been described in a rheumatology population and given such prominence in a PROM. Described as a replenishable resource that was essential to support physical and mental activity [16], the conceptual underpinnings of the WASTEd questionnaire confirmed energy as a necessary component of fatigue assessment, with the potential to be scored as its own subscale. Likewise, self-management and coping also emerged as essential, patient-derived components of patients’ fatigue experience and were thus incorporated as items within the measure. Although these are not new concepts to the experience of axSpA [5, 6, 16], the WASTEd represents the first time that such patient-important outcomes have been incorporated into fatigue assessment. Moreover, the WASTEd questionnaire incorporates an item on fatigue frequency, addressing a known gap in axSpA-fatigue measurement and thus supporting identification of patients with major fatigue (i.e. both frequent and severe) [4].

Developed using a transparent and methodologically robust process [13–15], the new fatigue and energy framework was derived from qualitative work with patients [16] and reviews of existing measures of fatigue and/or energy from both within [9, 21] and outside rheumatology [19, 20]. The distinction of energy as a separate but essential aspect of fatigue experience extends previous knowledge and might help to determine a patient’s ability, for example, to maintain their home axSpA-exercise regimens. The reviews of existing measures identified only one assessment of energy: the vitality subscale of the SF-36 [25]. Although widely used, measurement evidence is too limited to recommend its use for axSpA fatigue assessment [9].

Exploration of the developing measurement framework with separate groups of patients and health professionals also confirmed the content, relevance and acceptability of the developing PROM in capturing the fatigue outcomes that really matter, the severity of fatigue and energy, associated impact on daily life and ability of patients to self-manage [18]. This triad formed part of the analysis process in this study, making the impact of fatigue and energy the focus of the PROM. Evidence suggests that PROMs with clear conceptual underpinnings have high levels of face and content validity [11, 13, 15], which can enhance patient acceptability and clinical utility and improve responsiveness to important changes in health [31].

Although the numbers of patient participants in the focus groups were limited, the voice of patients was widely represented throughout the development process and further enhanced by the active involvement of our PRPs at all key stages (Supplementary Table S1, available at *Rheumatology Advances in Practice* online). Although all patient participants in stage 3 cognitive interviews were male, the contribution of the PRPs (4 female and 3 male) to the analysis ensured that a gendered view of the data was facilitated. A rigorous approach to all phases of the qualitative research is described, which involved patient, clinical and research experts participating in an iterative approach to item development and refinement. This process increases confidence that our multifaceted approach has minimized the risk that any patient-important outcomes have been omitted from the measurement framework. The involvement of health-care professionals in additional focus groups was essential to enhancing the clinical relevance of the model and is a further strength of the WASTEd.

The first-version, 30-item WASTEd has demonstrable face and content validity, underpinned by rigorous qualitative research and active patient involvement [16]. Future research will seek to administer the measure to a larger and more diverse UK-wide patient population (in terms of disease duration, disease activity and socio-demographic variables). Quantitative assessment, using modern psychometric theory, will inform item reduction towards a short-form set of items that best represents patient-reported fatigue. Further analysis will confirm the dimensionality of the fatigue and energy model, measurement reliability, construct validity and ability to detect change in fatigue. Such evidence is crucial to confirming the suitability of the developing PROM for use in research and clinical practice. This work highlights fatigue and energy as important and measurable patient-reported concepts in axSpA. It is an important step towards the availability of a high-quality, patient-derived, relevant and acceptable PROM.

## Supplementary Material

rkac027_Supplementary_Data

## References

[1] Raine C , Keat A. Axial spondyloarthritis. Medicine 2014;42:251–6.

[2] Boonen A , Braun J, van der Horst Bruinsma IE et al ASAS/WHO ICF Core Sets for ankylosing spondylitis (AS): how to classify the impact of AS on functioning and health. Ann Rheum Dis 2010;69:102–7. 19282309 10.1136/ard.2008.104117

[3] Packham J. Optimizing outcomes for ankylosing spondylitis and axial spondyloarthritis patients: a holistic approach to care. Rheumatology 2018;57:vi29–34. 30445484 10.1093/rheumatology/key200 PMC6238224

[4] Haywood KL , Packham JC, Jordan KP. Assessing fatigue in ankylosing spondylitis: the importance of frequency and severity. Rheumatology 2014;53:552–6. 24307290 10.1093/rheumatology/ket397

[5] Farren W , Goodacre L, Stigant M. Fatigue in ankylosing spondylitis: causes, consequences and self-management. Musculoskelet Care 2013;11:39–50. 10.1002/msc.1029 22825963

[6] Davies H , Brophy S, Dennis M et al Patient perspectives of managing fatigue in Ankylosing Spondylitis, and views on potential interventions: a qualitative study. BMC Musculoskelet Disord 2013;14:163. 23659344 10.1186/1471-2474-14-163 PMC3668149

[7] Sieper J , Rudwaleit M, Baraliakos X et al The Assessment of SpondyloArthritis international Society (ASAS) handbook: a guide to assess spondyloarthritis. Ann Rheum Dis 2009;68(Suppl 2):ii1–44. 19433414 10.1136/ard.2008.104018

[8] Garrett S , Jenkinson T, Kennedy LG et al A new approach to defining disease status in ankylosing spondylitis: the Bath Ankylosing Spondylitis Disease Activity Index. J Rheumatol 1994;21:2286–91. 7699630

[9] Pearson NA , Packham JC, Tutton E, Parsons H, Haywood KL. Assessing fatigue in adults with axial spondyloarthritis: a systematic review of the quality and acceptability of patient-reported outcome measures. Rheumatology advances in practice 2018;2:rky017. 31431965 10.1093/rap/rky017 PMC6649921

[10] Lasch KE , Marquis P, Vigneux M et al PRO development: rigorous qualitative research as the crucial foundation. Qual Life Res 2010;19:1087–96. 20512662 10.1007/s11136-010-9677-6 PMC2940042

[11] Bredart A , Marrel A, Abetz-Webb L, Lasch K, Acquadro C. Interviewing to develop Patient-Reported Outcome (PRO) measures for clinical research: eliciting patients’ experience. Health Qual Life Outcomes 2014;12:15. 24499454 10.1186/1477-7525-12-15 PMC3933509

[12] Haywood KL , de Wit M, Staniszewska S, Morel T, Salek S. Developing patient-reported and relevant outcome measures. In: Facey KM, Ploug Hansen H, Single ANV, eds. Patient involvement in health technology assessment. Singapore: Springer Singapore, 2017: 103–20. doi:10.1007/978-981-10-4068-9_9.

[13] Patrick DL , Burke LB, Gwaltney CJ, Leidy NK et al Content validity–establishing and reporting the evidence in newly developed patient-reported outcomes (PRO) instruments for medical product evaluation: ISPOR PRO good research practices task force report: part 1–eliciting concepts for a new PRO instrume. Value Heal J Int Soc Pharmacoeconomics Outcomes Res 2011;14:967–77. 10.1016/j.jval.2011.06.014 22152165

[14] US Food and Drug Administration. Guidance for industry: patient-reported outcome measures: use in medical product development to support labeling claims. Rockville: Department of Health and Human Services, Food and Drug Administration, Centre for Drug Evaluation and Research, 2009.

[15] Patrick DL , Burke LB, Gwaltney CJ, Leidy NK et al Content validity—establishing and reporting the evidence in newly developed patient-reported outcomes (PRO) instruments for medical product evaluation: ISPOR PRO good research practices task force report: part 2—assessing respondent understanding. Value Heal 2011;14:978–88. 10.1016/j.jval.2011.06.013 22152166

[16] Pearson NA , Tutton E, Martindale J et al Qualitative interview study exploring the patient experience of living with axial spondyloarthritis and fatigue: difficult, demanding and draining. BMJ Open 2022;12:e053958. 10.1136/bmjopen-2021-053958 PMC8883261 35217538

[17] Braun V , Clarke V. Using thematic analysis in psychology. Qual Res Psychol 2006;3:77–101.

[18] Sanderson TC , Hewlett SE, Flurey C et al The impact triad (severity, importance, self-management) as a method of enhancing measurement of personal life impact of rheumatic diseases. J Rheumatol 2011;38:191–4. 21285178 10.3899/jrheum.100700

[19] Whitehead L. The measurement of fatigue in chronic illness: a systematic review of unidimensional and multidimensional fatigue measures. J Pain Symptom Manage 2009;37:107–28. 19111779 10.1016/j.jpainsymman.2007.08.019

[20] Neuberger GB. Measures of fatigue: The Fatigue Questionnaire, Fatigue Severity Scale, Multidimensional Assessment of Fatigue Scale, and Short Form-36 Vitality (Energy/Fatigue) Subscale of the Short Form Health Survey. Arthritis Care Res 2003;49:S175–83.

[21] Hewlett S , Dures E, Almeida C. Measures of fatigue: Bristol Rheumatoid Arthritis Fatigue Multi-Dimensional Questionnaire (BRAF MDQ), Bristol Rheumatoid Arthritis Fatigue Numerical Rating Scales (BRAF NRS) for Severity, Effect, and Coping, Chalder Fatigue Questionnaire (CFQ), Checklist. Arthritis Care Res 2011;63:S263–86. 10.1002/acr.20579 22588750

[22] Rudwaleit M , van der Heijde D, Landewé R et al The development of Assessment of SpondyloArthritis international Society classification criteria for axial spondyloarthritis (part II): validation and final selection. Ann Rheum Dis 2009;68:777–83. 19297344 10.1136/ard.2009.108233

[23] Flesch R. A new readability yardstick. J Appl Psychol 1948;32:221–33. 18867058 10.1037/h0057532

[24] Hak T , van der Veer K, Jansen H. The Three-Step Test-Interview (TSTI): an observation-based method for pretesting self-completion questionnaires. Surv Res Methods 2008;2:143–150.

[25] Ware JEJ , Sherbourne CD. The MOS 36-item short-form health survey (SF-36). I. Conceptual framework and item selection. Med Care 1992;30:473–83. 1593914

[26] Yellen SB , Cella DF, Webster K, Blendowski C, Kaplan E. Measuring fatigue and other anemia-related symptoms with the Functional Assessment of Cancer Therapy (FACT) measurement system. J Pain Symptom Manage 1997;13:63–74. 9095563 10.1016/s0885-3924(96)00274-6

[27] Van Tubergen A , Debats I, Ryser L et al Use of a numerical rating scale as an answer modality in ankylosing spondylitis-specific questionnaires. Arthritis Rheum 2002;47:242–8. 12115152 10.1002/art.10397

[28] Lerdal A , Gay CL, Aouizerat BE, Portillo CJ, Lee KA. Patterns of morning and evening fatigue among adults with HIV/AIDS. J Clin Nurs 2011;20:2204–16. 21752119 10.1111/j.1365-2702.2011.03751.x PMC3136915

[29] Aouizerat BE , Gay CL, Lerdal A, Portillo CJ, Lee KA. Lack of energy: an important and distinct component of HIV-related fatigue and daytime function. J Pain Symptom Manage 2013;45:191–201. 22917712 10.1016/j.jpainsymman.2012.01.011 PMC3547130

[30] Jacobson J , Ju A, Baumgart A et al Patient perspectives on the meaning and impact of fatigue in hemodialysis: a systematic review and thematic analysis of qualitative studies. Am J Kidney Dis 2019;74:179–92. 30955947 10.1053/j.ajkd.2019.01.034

[31] Guyatt GH , Bombardier C, Tugwell PX. Measuring disease-specific quality of life in clinical trials. CMAJ 1986;134:889–95. 3955482 PMC1490966

