# Supplementary material for: Development of the Warwick Axial Spondyloarthritis faTigue and Energy questionnaire (WASTEd)—a new patient-reported outcome measure
Source: Rheumatol Adv Pract. 2022 Apr 4;6(1):rkac027. doi: 10.1093/rap/rkac027 (PMC9021732; doi:10.1093/rap/rkac027)
Supplement: rkac027_Supplementary_Data [file rkac027_supplementary_data.docx]

# Supplementary Table S1: GRIPP2 short form reporting checklist of PPI involvement (1)

| **Section and topic** | **Item** |
| --- | --- |
| Aim | To co-produce a new patient-reported outcome measure of fatigue in axSpA |
| Methods | Five patient partners worked collaboratively with the research team. Two of these members were involved in the study from its conception and were listed as co-applicants on the grant submission. Patient partners were involved using different methods, including face-to-face meetings, emailing, telephone calls and were sent study summaries to remain current with study activity.  Patient partners assisted the research team in all stages of developing and refining the WASTEd. Their involvement spanned the design of study materials (ethics applications, interview schedules), the analysis of qualitative data to produce a conceptual model of axSpA fatigue, the development of verbal prompts for pretesting interviews, and group analysis and interpretation of pretesting interview data to refine the WASTEd. Additionally, patient partners helped the research team to craft items for the questionnaire, ensuring comprehensiveness, and comprehensibility of the new PROM. Further input included reviewing the presentation of the WASTEd (formatting, layout, and overall presentation). |
| Study results | Patient partners made a range of contributions to the study, including:   - Interpreting interview findings and contributing to the revision of the measurement framework, with a particular role in further highlighting the distinction between fatigue and energy - Identifying problematic items or domains (in terms of relevance or comprehensibility) and crafting suitable verbal prompts to facilitate details assessment in pretesting interviews - Co-analysing (with NP and JM) the findings from pretesting interviews to reach a group decision on how to revise and improve the WASTEd. - Providing input regarding how the WASTEd is presented to improve accessibility for patients. Notable modifications included reducing the number of items per page to between four and six and providing a brief instructions sheet at the front of the WASTEd. - Reviewing and providing comments/ feedback on the study manuscript. |
| Discussion and conclusions | Patient involvement in this study was central. Patient partners helped to shape the study and conceive the idea as part of the funding application. Throughout the process of developing the WASTEd, patient partners have been vocal advocates for the need to develop a PROM that is relevant, accessible, and comprehensive. Their effectiveness as a group may be due to the high-quality training they receive as part of the team at Wrightington Hospital, coupled with their experience working on other studies as patient partners.  Regular involvement through face-to-face meetings ensured patient partners had a space to share their views safely, with time to articulate their views. Group discussions often followed and often resulted in the group reaching consensus on suggestions to improve the WASTEd. This process was facilitated by the acquisition of adequate funding to support their involvement and any related activities for the study. |
| Reflections/ critical perspective | Patient partners were involved as closely and frequently as was feasible within the study. Whilst methods such as emailing and sharing summaries were effective for maintaining contact and keeping members current with study progress, they were not used for decision-making whilst a study was *in vivo*. Such methods may be more effective for group decision-making on smaller points of consideration, such as revising an interview schedule. |

GRIPP: Guideline for Reporting Involvement of Patients and the Public; PPI: patient and public involvement.

# Supplementary Table S2: Items identified per domain of the WASTEd measurement framework – the results of PROM mapping

| **Conceptual domains** | **Subdomains** | **Components** | **PROMs used** | **Identified items** |
| --- | --- | --- | --- | --- |
| **Symptoms** | Fatigue | Frequency; Severity; Duration | **BRAF-MDQ** *(items 1-3)*  **BASDAI** *(item 1)*  **MAF** *(items 2 and 15)*  **FACIT-F** *(item 3)*  **SF-36v2 VT** *(items 9g and 9i)* | **Frequency**  *BRAF-MDQ*: “How many days have you experienced fatigue?”  *MAF*: “Over the past week, how often have you been fatigued?”  *SF-36v2 VT*: “Did you feel worn out?”  *SF-36v2 VT*: “Did you feel tired?”  **Severity**  *BRAF-MDQ*: “How severe is the fatigue that you have experienced?”  *BASDAI*: “How would you describe the overall level of fatigue/tiredness you have experienced?”  *MAF*: “How severe is the fatigue which you have been experiencing?”  *FACIT-F*: “I feel listless (‘washed out’)”  **Duration**  *BRAF-MDQ*: “How long, on average, does each spell of fatigue last?” |
|  | Low energy | Frequency; Severity; Duration | **BRAF-MDQ** *(items 1-3)*  **SF-36v2 VT** (item 9e)  **FACIT-F** *(item 7)* | *BRAF-MDQ*: Modification of items 1 to 3 to reflect energy  **Level of energy**  *SF-36v2 VT*: “Did you have a lot of energy?”  *FACIT-F*: “I have energy” |
| **Impact** | Cognitive | Impact – reading; talking; memory; ability to concentrate | **BRAF-MDQ** *(item 15)*  **CFS** *(items 8 and 9)*  **MFI-20** *(item 11)*  **MFSI-SF** *(item 1)* | **Talking**  *CFS*: “do you make slips of the tongue when speaking?”  **Concentration**  *BRAF-MDQ*: “Has fatigue made it difficult to concentrate?”  *CFS*: “do you have difficulties concentrating?”  **Memory**  *CFS*: “How is your memory?”  *MFI-20*: “I can concentrate well.”  *MFSI-SF*: “I have trouble remembering things” |
|  | Physical | Impact; self-care - eating; exercise | **MAF** *(items 5 and 14)*  **FACIT-F** *(item an12)*  **FSS** *(item 2)* | **Self-care**  *MAF*: “In the past week, to what degree has fatigue interfered with your ability to cook?”  *FACIT-F*: “I am too tired to eat”  **Exercise**  *MAF*: “In the past week, to what degree has fatigue interfered with your ability to exercise, other than walking?”  *FSS*: “Exercise brings on my fatigue” |
|  | Social | Impact/ interference on participation – usual work; job; leisure activities (family or friends); | **BRAF-MDQ** *(items 8, 10)*  **MFI-20** *(item 15)*  **FSS** *(item 9)*  **BFI** *(item 4f)*  **MAF** *(item 11)*  **FACIT-F** *(item An7)* | **Interference (generic)**  *FSS*: “Fatigue interferes with my work, family, or social life”  *BFI*: “Circle the one number that describes how, during the past 24 hours, fatigue has interfered with your relations with other people”  **Usual activity**  *FACIT-F*: “I am able to do my usual activities”  **Leisure activities**  *MAF*: “In the past week, to what degree has fatigue interfered with your ability to engage in leisure and recreational activity?”  **Social participation**  *BRAF-MDQ*: “Have you cancelled plans because of fatigue? e.g., plans to go out, or do jobs around the home or garden”  *BRAF-MDQ*: “Have you avoided making plans because of fatigue? e.g., plans to go out, or do jobs around the home or garden”  *MFI-20*: “I have a lot of plans.” |
| **Sleep** |  | Difficulty getting to sleep; disturbed sleep; feeling when awake (refreshed?) |  | **No items identified** |
| **Psychological/ emotional wellbeing** | Mood | Depression; Low mood; feeling down; Losing control; helplessness | **BRAF-MDQ** *(items 17 and 20)*  **BFI** *(item 4b)*  **MFSI-SF** *(items 3 and 21)* | **Control**  *BRAF-MDQ*: Have you felt you have less control in areas of your life because of fatigue?  **Depression**  *BRAF-MDQ*: “Have you felt down or depressed because of fatigue?”  *BFI*: “Circle the one number that describes how, during the past 24 hours, fatigue has interfered with your mood.”  *MFSI-SF*: “I feel upset”  *MFSI-SF*: “I feel depressed” |
|  | Anxiety/ worrying | Worry; Dread; Frustration; Stress; Unpredictable | **MFI-20** *(item 9)*  **FACIT-F** *(item An15)* | **Dread**  *MFI-20*: “I dread having to do things.”  **Frustration**  *FACIT-F*: “I am frustrated by being too tired to do the things I want to do” |
|  | Sense of self | Guilt – letting others down; Embarrassment – falling asleep in social situations; helplessness | **BRAF-MDQ** *(item 18)* | *BRAF-MDQ*: “Have you felt embarrassed because of fatigue?” |
|  | Self-isolation | Pushing others away; hiding the fatigue; invisible illness |  | **No items identified** |
| **Self-management** | Achieving balance | Avoiding overexertion; avoiding underactivity; dealing with new needs (coping and adapting); learnt expertise; taking a flexible approach; lost – don’t know what to do. | **MFI-20** *(items 2 and 8)* | **Exertion and activity**  *MFI-20*: “Physically, I feel only able to do a little”  *MFI-20*: "Physically I can take on a lot.” |
|  | Energy management | Difficulty starting things; Re-energising (coffee/sugar, napping); declining events to avoid wipe-out; feeling drained | **CFS** *(item 4)*  **FACIT-F** *(item An3)* | **Difficulty starting things**  *CFS*: “do you have problems starting things?”  *FACIT-F*: “I have trouble starting things because I am tired” |
|  | Support | Delegating responsibilities (family, friends, colleagues) | **FACIT-F** *(item An14)* | *FACIT-F*: “I need help doing my usual activities” |

BASDAI: Bath Ankylosing Spondylitis Disease Activity Index; BFI: Brief Fatigue Inventory; BRAF-MDQ; CFS: Chalder Fatigue Scale; FACIT-F: Functional Assessment of Chronic Illness Therapy – Fatigue; FSS: Fatigue Severity Score; MAF: Multidimensional Assessment of Fatigue; MFI-20: Multidimensional Fatigue Inventory; MFSI-SF: Multidimensional Fatigue Symptom Inventory – Short Form; SF-36v2 VT: Short-Form 36-item Health Survey.

# Supplementary Table S3: Summary of identified changes from round 1 cognitive interviews, research team review and PRP group review

| **#** | **Question** | **Changes proposed** | | |
| --- | --- | --- | --- | --- |
|  |  | *Cognitive interviews (R1)* | *Research team (R1)* | *PRP group (R1)* |
| 1 | How often have you felt fatigued? | - | - | - |
| 2 | How severe was the fatigue? | Response options  “a lot” changed to “very” | - | *Response options*  “Not at all” changed to “not at all severe”  “extremely” changed to “extremely severe” |
| 3 | How much energy have you had? | Response options  “Not at all” changed to “None” | - | - |
| 4 | How often have you felt drained? | Needs clarity | Add clarity – “… drained of energy” | *Response options*  “a little” changed to “rarely” |
| 5 | To what extent have your energy levels interfered with your ability to chat to other people? (e.g., friends, family, work colleagues) | *Change syntax*  “Have you found it difficult to chat to other people because of fatigue?” | *Language*  Change the word ‘chat’ to ‘hold a conversation’ to reduce vagueness | *Changed to fatigue*  “Hold a conversation” changed to “engage in conversations” |
| 6 | To what extent have your energy levels interfered with your ability to remember things? (e.g., being more forgetful than normal) | *Split item* | - | *Delete this item* |
| 6a | To what extent has fatigue interfered with your ability to remember things? | *New version of item 6a* | - | *Retain this item* |
| 7 | To what extent have your energy levels interfered with your ability to concentrate? (e.g., reading, writing, jigsaws, crosswords) | *Split item* | Updated examples: reading removed | *Energy version removed*  Examples changed to “driving, puzzles and electronic games” |
| 7a | To what extent has fatigue interfered with your ability to concentrate? (e.g., reading, follow a film or TV program) | *New version of item 7a* | - | - |
| 8 | To what extent have your energy levels interfered with your ability to take care of yourself? (e.g., cooking, eating well throughout the day) | - | - | *Example*  Prefer ‘personal care’ than showering or bathing |
| 9 | To what extent have your energy levels interfered with your ability to do physical things? | - | Examples added | *Vague*  Physical “things” not precise enough, “activities” a better word |
| 10 | To what extent have your energy levels interfered with your ability to take part in more demanding exercise of moderate intensity? (e.g., going for a walk, swimming) | - | - | *Wording changed*  Change part to “more demanding physical activities of moderate intensity?” |
| 11 | To what extent have your energy levels interfered with your ability to do your usual work? (e.g., at work or at home) | - | - | *Probe*  Is this similar to item 10? |
| 12 | To what extent would, or have your energy levels interfered with your ability to do the things you enjoy? (e.g., hobbies, leisure activities) | *Split item* | - | *Delete this item* |
| 12a | To what extent would, or has fatigue interfered with your ability to do the things you enjoy? (e.g., listening to music) | - | - | *Retain this item* |
| 13 | Because of fatigue would you, or have you avoided making plans? | *Split item* | - | *Delete this item* |
| 13a | Would you, or have you avoided making plans in case you might not have the energy to do them? | *New version of item 13a* | - | *Retain this item* |
| 14 | Because of fatigue would you, or have you cancelled plans? (e.g., a trip somewhere, holiday) | *Split item* | *Delete this item* | *Delete this item* |
| 14a | Would you, or have you cancelled plans because you did not have the energy to do them? | *New version of item 14a* | *Retain this item* | *Retain this item* |
| 15 | Because of fatigue would you, or have you turned down invitations? (e.g., to meet a friend, socialise) | *Split item* | *Delete this item* | - |
| 15a | Would you, or have you turned down invitations because you did not have the energy to go? (e.g., to meet a friend, socialise) | *New version of item 15a* | *Retain* *this item* | - |
| 16 | On average, what was the quality of your sleep? | *Performing poorly* | *Delete this item* | *Delete this item* |
| 17 | Have you woken up ready to face the day? | *Performing poorly* | *Delete this item* | *Delete this item* |
| 18 | Has fatigue made you feel downhearted? | *Reword*  “Has you felt downhearted because of fatigue” | *Examples added* | - |
| 19 | Has fatigue made you feel like you have less control in your life? | *Reword*  “Have you felt like you have less control in your life because of fatigue?” | Reworded | *Probe*  Is this lots of things other than fatigue? |
| 20 | Has fatigue left you feeling worried? (e.g., about being able to cope)" | *Reword*  “Have you felt worried about being able to get through the week, because of fatigue?” | *Example removed* | - |
| 21 | Has fatigue left you feeling frustrated? | *Reword*  “Have you felt frustrated because of fatigue?” | - | - |
| 22 | Has fatigue left you feeling overwhelmed? | Delete this item | Delete this item | - |
| 23 | Have you preferred to be alone because of fatigue? | - | - | - |
| 24 | Have you felt like the fatigue is invisible to others? (e.g., people do not seem to understand) | *Delete this item* | *Delete this item* | - |
| 25 | I have struggled to find the balance to maintain my energy levels (e.g., doing too little or too much). | *Create two replacement items and delete this item* | *-* | - |
| 25 | I have struggled to maintain my energy levels because I have done too little. | - | *Delete this item* | - |
| 25a | I have struggled to maintain my energy levels because I have done too much. | - | *Retain this item* | - |
| 26 | I feel I have coped well with fatigue. | *Introduce new item*  *Reword to manage*  “I have been able to manage my fatigue.” | - | - |
| 26a | I have been able to manage my energy levels. | - | - | - |
| 27 | Because of your energy levels, have you found it physically difficult to start, or finish doing things? | *Create two replacement items and delete this item* | *Replacement item for 27, a and b*  Have you lacked physical energy? | - |
| 27a | Because of your energy levels, have you found it physically difficult to start doing things? | - | *Delete this item* | - |
| 27b | Because of your energy levels, have you found it physically difficult to finish doing things? | - | *Delete this item* | - |
| 28 | Because of your energy levels, have you found it mentally difficult to start, or finish doing things? | *Create two replacement items and delete this item* | *Replacement item for 28, a and b*  Have you lacked mental energy? | - |
| 28a | Because of your energy levels, have you found it mentally difficult to start doing things? | - | *Delete this item* | - |
| 28b | Because of your energy levels, have you found it mentally difficult to finish doing things? | - | *Delete this item* | - |
| 29 | Have you run out of energy quickly and needed to take a break? (e.g., have a nap or rest) | - | - | - |
| 30 | Are you using stimulants to boost your energy? (e.g., coffee, sugary drinks or foods) | - | Remove item | - |
| 31 | Because of your fatigue, would you, or have you had to ask friends and/or family to do things for you? | *Example added*  “e.g., run errands, do things around the house” | - | *Probe*  Is this about dependence? (Language from team meeting) |
| 32 | Would you, or have you been able to share your experience or feelings of fatigue with someone? (e.g., friends, family, healthcare professional) | - | *Delete this item* | - |

PRP: patient research partner.

# Supplementary Table S4: Summary of identified changes from round 2 cognitive interviews, research team review and PRP group review

| **#** | **Question** | **Changes proposed** | | |
| --- | --- | --- | --- | --- |
|  |  | *Cognitive interviews (R2)* | *Research team (R2)*^[[1]](#footnote-1)^ | *PRP group (R2)* |
| *Section 1: Fatigue* | | | | |
| F1 | How often have you felt fatigued? | *Response options*  Change “always” to “everyday” | - | - |
| F2 | How severe was the fatigue? | - | - | *Language*  Change “the fatigue” to “your fatigue” |
| F3 | Has your fatigue made it difficult to remember things? (e.g., being more forgetful than normal) | *Response options*  Change “completely” to “extremely” | - | - |
| F4 | Has your fatigue made it difficult to concentrate on demanding tasks? (e.g., driving, puzzles, electronic games) | *Language*  “demanding” carries physical connotations – discuss with team and PRP | - | - |
| F5 | Have you found it difficult to engage in conversations with other people because of your fatigue? (e.g., friends, family, work colleagues) | - | - | - |
| F6 | Has your fatigue made it difficult to do the things you enjoy? (e.g., listening to music, watching a TV programme) | - | - | - |
| F7 | Have you felt downhearted because of your fatigue? (e.g., feeling low or down) | - | - | - |
| F8 | Have you felt you have less control in your life because of your fatigue? | *Delete this item* | - | - |
| F9 | Have you felt worried because of your fatigue? | - | - | - |
| F10 | Have you felt frustrated because of your fatigue? | - | - | - |
| F11 | Have you preferred to be left alone because of your fatigue? (e.g., not interacting with friends or relatives) | *Language*  Not a “preference”  *Reworded*  “Have you felt the need to be left alone because of your fatigue? (e.g., not interacting with friends or relatives)” | - | - |
| F12 | I feel I have been able to manage my fatigue. | *Additional item identified* | - | - |
| F12a | I feel I have been able to cope with my fatigue. | *New item (reintroduced)* | *Flagged*  Is this *really* different? | *Retain this item* |
| F13 | Have you been more dependent on others (e.g., friends or family) because of your fatigue? (e.g., to run errands, do things around the house) | *Example*  Include “emotional support” | - | *Example*  Remove “emotional support” |
| Section 2: Energy | | | | |
| E1 | How much energy have you had? | - | - | - |
| E2 | How often have you felt drained of energy? | - | - | - |
| E3 | Have your energy levels made it difficult to take care of yourself? (e.g., cooking, personal care) | - | - | *Example*  Change “cooking” to “showering or “brushing your teeth” |
| E4 | Have your energy levels made it difficult to do physical activities? (e.g., light housework, making something to eat or drink) | - | - | - |
| E5 | Have your energy levels made it difficult to take part in more demanding physical activities of moderate intensity? (e.g., going for a walk, swimming) | - | - | *Example*  Change “swimming” to “gardening” |
| E6 | Have your energy levels made it difficult to do your usual work? (e.g., at work or at home) | *Repetition with E7*  Discuss with team and PRP | - | - |
| E7 | Have your energy levels made it difficult to do the things you enjoy? (e.g., hobbies, leisure activities) | *Repetition with E6*  Discuss with team and PRP | - | - |
| E8 | Have your energy levels made it difficult to make plans? | - | - | - |
| E9 | Have your energy levels cause you to cancel plans? | - | - | - |
| E10 | Have your energy levels made it difficult to keep to your plans? | - | - | - |
| E11 | Have you 'turned down' invitations because of your energy levels? (e.g., to meet a friend, socialise) | - | - | - |
| E12 | I have struggled to maintain my energy levels because I have done too much. | - | - | - |
| E13 | I feel I have been able to manage my energy levels. | - | - | - |
| E13a | I feel I have been able to cope with my energy levels | - | - | - |
| E14 | Have you lacked physical energy? | - | - | - |
| E15 | Have you lacked mental energy? | - | - | - |
| E16 | Have you run out of energy quickly and needed to take a break? (e.g., have a nap or rest) | - | - | - |

PRP: patient research partner.

# Supplementary Data S1: A copy of the long-form WASTEd following pretesting interview revisions

**
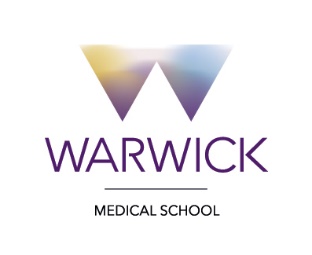

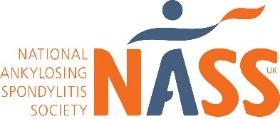
**

**Warwick Axial Spondyloarthritis Fatigue and Energy Description scale (WASTED)**

Instructions

We would like to know how your fatigue and energy levels associated with your Axial Spondyloarthritis (axSpA) have affected you, ***on average****,* over the ***past 7-days***.

We understand that your fatigue and energy levels may have changed day-to-day, but we would like you to answer the questions about how you have been feeling ***on average*** over the ***past 7-days***. This will let us understand how you feel and are affected by your axSpA-fatigue and help us decide how best we can support you.

The questionnaire is separated into two sections:

**Section 1 – Fatigue:** here, we ask 14 questions about your experience of fatigue associated with your axSpA and how it has affected you, ***on average, over the past 7-days***.

**Section 2 – Energy:** here, we ask 16 questions about your energy levels associated with your axSpA and how they have affected you, ***on average, over the past 7-days***.

**Please read each question carefully and answer each question with a single cross.**

**Section 1: Fatigue**

Please read the following statement before completing this section.

**Fatigue:** Everyone gets tired or even worn-out at times, but after a few good night’s rest they usually feel refreshed. It is known that people with this condition experience fatigue which is not like normal tiredness. Fatigue can last for weeks at a time and no amount of sleep or rest will relieve it.

_________________________________________

On **AVERAGE** over the **PAST 7-DAYS**

|  | **How often have you felt fatigued?** | | | | |
| --- | --- | --- | --- | --- | --- |
|  | |  |  |  |  |
|  | |  |  |  |  |
| Not at all | | Rarely | Sometimes | Often | All the time |

|  | **How severe was your fatigue?** | | | | |
| --- | --- | --- | --- | --- | --- |
|  | |  |  |  |  |
|  | |  |  |  |  |
| Not at all severe | | A little | Moderately | Very | Extremely severe |

|  | **Has** **your fatigue made it difficult to remember things? (e.g. being more forgetful than normal)** | | | | |
| --- | --- | --- | --- | --- | --- |
|  | |  |  |  |  |
|  | |  |  |  |  |
| Not at all difficult | | A little | Moderately | Very | Extremely difficult |

|  | **Has your fatigue made it difficult to concentrate on demanding tasks? (e.g. driving, puzzles, electronic games)** | | | | |
| --- | --- | --- | --- | --- | --- |
|  | |  |  |  |  |
|  | |  |  |  |  |
| Not at all difficult | | A little | Moderately | Very | Extremely difficult |

On **AVERAGE** over the **PAST 7-DAYS**

|  | **Have you found it difficult to engage in conversations with other people because of your fatigue? (e.g. friends, family, work colleagues)** | | | | |
| --- | --- | --- | --- | --- | --- |
|  | |  |  |  |  |
|  | |  |  |  |  |
| Not at all difficult | | A little | Moderately | Very | Extremely difficult |

|  | **Has your fatigue made it difficult to do the things you enjoy? (e.g. listening to music, watching a TV programme)** | | | | |
| --- | --- | --- | --- | --- | --- |
|  | |  |  |  |  |
|  | |  |  |  |  |
| Not at all difficult | | A little | Moderately | Very | Extremely difficult |

|  | **Have you felt downhearted because of your fatigue? (e.g. feeling low or down)** | | | | |
| --- | --- | --- | --- | --- | --- |
|  | |  |  |  |  |
|  | |  |  |  |  |
| Not at all downhearted | | A little | Moderately | Very | Completely downhearted |

|  | **Has your fatigue made it difficult to be in control of your life?** | | | | |
| --- | --- | --- | --- | --- | --- |
|  | |  |  |  |  |
|  | |  |  |  |  |
| Not at all difficult | | A little | Moderately | Very | Extremely difficult |

|  | **Have you felt worried because of your fatigue?** | | | | |
| --- | --- | --- | --- | --- | --- |
|  | |  |  |  |  |
|  | |  |  |  |  |
| Not at all worried | | A little | Moderately | Very | Extremely worried |

On **AVERAGE** over the **PAST 7-DAYS**

|  | **Have you felt frustrated because of your fatigue?** | | | | |
| --- | --- | --- | --- | --- | --- |
|  | |  |  |  |  |
|  | |  |  |  |  |
| Not at all frustrated | | A little | Moderately | Very | Extremely frustrated |

|  | **Have you felt the need to be left alone because of your fatigue? (e.g. not interacting with friends or relatives)** | | | | |
| --- | --- | --- | --- | --- | --- |
|  | |  |  |  |  |
|  | |  |  |  |  |
| Not at all | | A little | Sometimes | A lot | All the time |

|  | **I feel I have been able to manage my fatigue.** | | | | |
| --- | --- | --- | --- | --- | --- |
|  | |  |  |  |  |
|  | |  |  |  |  |
| Not at all | | A little | Sometimes | A lot | All the time |

|  | **I feel I have been able to cope with my fatigue.** | | | | |
| --- | --- | --- | --- | --- | --- |
|  | |  |  |  |  |
|  | |  |  |  |  |
| Not at all | | A little | Sometimes | A lot | All the time |

|  | **Have you been more dependent on others (e.g. friends or family) because of your fatigue? (e.g. to run errands, do things around the house for you).** | | | | |
| --- | --- | --- | --- | --- | --- |
|  | |  |  |  |  |
|  | |  |  |  |  |
| Not at all | | A little | Sometimes | A lot | All the time |

**Section 2: Energy levels**

Please read the following statement before completing this section.

**Energy:** Everyone usually has the energy levels to do things in their day, but with this condition it could be a real struggle to find that ‘get up and go’ to do the things you want or need to do. You may feel ‘drained’ and need to stop for a quick rest which might help you generate some energy.

_________________________________________

On **AVERAGE** over the **PAST 7-DAYS**

|  | **How much energy have you had?** | | | | |
| --- | --- | --- | --- | --- | --- |
|  | |  |  |  |  |
|  | |  |  |  |  |
| No energy | | A little | Moderate amount | A lot | Full of energy |

|  | **How often have you felt drained of energy?** | | | | |
| --- | --- | --- | --- | --- | --- |
|  | |  |  |  |  |
|  | |  |  |  |  |
| Not at all | | Rarely | Sometimes | Often | All the time |

|  | **Have your energy levels made it difficult to take care of yourself? (e.g. personal care, showering, brushing your teeth)** | | | | |
| --- | --- | --- | --- | --- | --- |
|  | |  |  |  |  |
|  | |  |  |  |  |
| Not at all difficult | | A little | Moderately | Very | Completely difficult |

|  | **Have your energy levels made it difficult to do every day activities? (e.g. light housework, making something to eat or drink)** | | | | |
| --- | --- | --- | --- | --- | --- |
|  | |  |  |  |  |
|  | |  |  |  |  |
| Not at all difficult | | A little | Moderately | Very | Extremely difficult |

On **AVERAGE** over the **PAST 7-DAYS**

|  | **Have your energy levels made it difficult to take part in more demanding physical activities? (e.g. going for a walk, gardening)** | | | | |
| --- | --- | --- | --- | --- | --- |
|  | |  |  |  |  |
|  | |  |  |  |  |
| Not at all difficult | | A little | Moderately | Very | Extremely difficult |

|  | **Have your energy levels made it difficult to do your usual work? (e.g. at work or at home)** | | | | |
| --- | --- | --- | --- | --- | --- |
|  | |  |  |  |  |
|  | |  |  |  |  |
| Not at all difficult | | A little | Moderately | Very | Extremely difficult |

|  | **Have your energy levels made it difficult to do the things you enjoy? (e.g. hobbies, leisure activities)** | | | | |
| --- | --- | --- | --- | --- | --- |
|  | |  |  |  |  |
|  | |  |  |  |  |
| Not at all difficult | | A little | Moderately | Very | Extremely difficult |

|  | **Have your energy levels made it difficult to make plans?** | | | | |
| --- | --- | --- | --- | --- | --- |
|  | |  |  |  |  |
|  | |  |  |  |  |
| Not at all difficult | | A little | Moderately | Very | Extremely difficult |

|  | **Have your energy levels caused you to cancel plans?** | | | | |
| --- | --- | --- | --- | --- | --- |
|  | |  |  |  |  |
|  | |  |  |  |  |
| Not at all | | A little | Sometimes | A lot | All the time |

On **AVERAGE** over the **PAST 7-DAYS**

|  | **Have your energy levels made it difficult to keep to your plans?** | | | | |
| --- | --- | --- | --- | --- | --- |
|  | |  |  |  |  |
|  | |  |  |  |  |
| Not at all difficult | | A little | Moderately | Very | Extremely difficult |

|  | **Have you ‘turned down’ invitations because of your energy levels? (e.g. to meet a friend, socialise)** | | | | |
| --- | --- | --- | --- | --- | --- |
|  | |  |  |  |  |
|  | |  |  |  |  |
| Not at all | | A little | Sometimes | A lot | All the time |

|  | **I have difficulty maintaining my energy levels when I have done too much.** | | | | |
| --- | --- | --- | --- | --- | --- |
|  | |  |  |  |  |
|  | |  |  |  |  |
| Not at all difficult | | A little | Sometimes | A lot | Extremely difficult |

|  | **I feel I have been able to manage my energy levels.** | | | | |
| --- | --- | --- | --- | --- | --- |
|  | |  |  |  |  |
|  | |  |  |  |  |
| Not at all | | A little | Moderately | A lot | Completely |

|  | **Have you lacked physical energy?** | | | | |
| --- | --- | --- | --- | --- | --- |
|  | |  |  |  |  |
|  | |  |  |  |  |
| Not at all | | A little | Sometimes | A lot | All the time |

On **AVERAGE** over the **PAST 7-DAYS**

|  | **Have you lacked mental energy?** | | | | | | | | |
| --- | --- | --- | --- | --- | --- | --- | --- | --- | --- |
|  | |  | |  | |  | |  | |
|  | | |  | |  | |  | |  |
| Not at all | | | A little | | Sometimes | | A lot | | All the time |

|  | **Have you run out of energy suddenly and needed to take a break? (e.g. have a nap or rest)** | | | | |
| --- | --- | --- | --- | --- | --- |
|  | |  |  |  |  |
|  | |  |  |  |  |
| Not at all | | A little | Sometimes | A lot | All the time |

# References

1. Staniszewska S, Brett J, Simera I, Seers K, Mockford C, Goodlad S, et al. GRIPP2 reporting checklists: tools to improve reporting of patient and public involvement in research. BMJ [Internet]. 2017 Aug 2;358:j3453. Available from: http://www.bmj.com/content/358/bmj.j3453.abstract

1. Meeting predominantly focused on response options which were standardised, with anchors added throughout. F = Fatigue item; E = Energy item. [↑](#footnote-ref-1)
